# Characterisation of *iunH* gene knockout strain from *Mycobacterium tuberculosis*

**DOI:** 10.1590/0074-02760160462

**Published:** 2017-03

**Authors:** Anne Drumond Villela, Valnês da Silva Rodrigues, Antônio Frederico Michel Pinto, Priscila Lamb Wink, Zilpa Adriana Sánchez-Quitian, Guilherme Oliveira Petersen, Maria Martha Campos, Luiz Augusto Basso, Diógenes Santiago Santos

**Affiliations:** 1 Pontifícia Universidade Católica do Rio Grande do Sul, Centro de Pesquisas em Biologia Molecular e Funcional, Instituto Nacional de Ciência e Tecnologia em Tuberculose, Porto Alegre, RS, Brasil; 2 Pontifícia Universidade Católica do Rio Grande do Sul, Programa de Pós-Graduação em Medicina e Ciências da Saúde, Porto Alegre, RS, Brasil; 3 Pontifícia Universidade Católica do Rio Grande do Sul, Instituto de Toxicologia e Farmacologia, Porto Alegre, RS, Brasil; 4 Pontifícia Universidade Católica do Rio Grande do Sul, Programa de Pós-Graduação em Biologia Celular e Molecular, Porto Alegre, RS, Brasil

**Keywords:** *iunH* gene, nucleoside hydrolase, gene knockout, Mycobacterium tuberculosis

## Abstract

**BACKGROUND:**

Tuberculosis (TB) is an infectious disease caused mainly by the bacillus *Mycobacterium tuberculosis*. The better understanding of important metabolic pathways from *M. tuberculosis* can contribute to the development of novel therapeutic and prophylactic strategies to combat TB. Nucleoside hydrolase (MtIAGU-NH), encoded by *iunH* gene (Rv3393), is an enzyme from purine salvage pathway in *M. tuberculosis*. MtIAGU-NH accepts inosine, adenosine, guanosine, and uridine as substrates, which may point to a pivotal metabolic role.

**OBJECTIVES:**

Our aim was to construct a *M. tuberculosis* knockout strain for *iunH* gene, to evaluate in vitro growth and the effect of *iunH* deletion in *M. tuberculosis* in non-activated and activated macrophages models of infection.

**METHODS:**

A *M. tuberculosis* knockout strain for *iunH* gene was obtained by allelic replacement, using pPR27*xylE* plasmid. The complemented strain was constructed by the transformation of the knockout strain with pNIP40::*iunH*. MtIAGU-NH expression was analysed by Western blot and LC-MS/MS. In vitro growth was evaluated in Sauton’s medium. Bacterial load of non-activated and interferon-γ activated RAW 264.7 cells infected with knockout strain was compared with wild-type and complemented strains.

**FINDINGS:**

Western blot and LC-MS/MS validated *iunH* deletion at protein level. The *iunH* knockout led to a delay in *M. tuberculosis* growth kinetics in Sauton’s medium during log phase, but did not affect bases and nucleosides pool in vitro. No significant difference in bacterial load of knockout strain was observed when compared with both wild-type and complemented strains after infection of non-activated and interferon-γ activated RAW 264.7 cells.

**MAIN CONCLUSION:**

The disruption of *iunH* gene does not influence *M. tuberculosis* growth in both non-activated and activated RAW 264.7 cells, which show that *iunH* gene is not important for macrophage invasion and virulence. Our results indicated that MtIAGU-NH is not a target for drug development.

Tuberculosis (TB) is an infectious disease caused mainly by the bacillus *Mycobacterium tuberculosis*, and remains one of the world’s deadliest contagious diseases. TB incidence is slowly declining each year, however, novel drugs and vaccines are urgently needed to stop global transmission and to prevent the development of drug-resistant strains (WHO 2016). The better understanding of important metabolic pathways from *M. tuberculosis* can contribute to the development of novel therapeutic and prophylactic strategies to combat TB. Purine nucleotides in *M. tuberculosis* may be formed *de novo* from simple precursors, or may be obtained by the salvage pathway from preformed purine bases and nucleosides. While the *de novo* pathway is a high energy demanding process, which involves up to 11 enzymatic steps, the salvage pathway might be the main source to maintain the nucleotide pool under conditions of low energy availability or rapid multiplication (Ducati et al. 2011). Nucleoside hydrolase (MtIAGU-NH), encoded by *iunH* gene (Rv3393, Gene ID: 887625), is an important enzyme from the purine salvage pathway in *M. tuberculosis*, which catalyses the irreversible hydrolysis of N-glycosidic bond of ribonucleosides forming a-D-ribose and the corresponding base (Wink et al. 2013). MtIAGU-NH was shown to have broad substrate specificity, accepting inosine, adenosine, guanosine, and uridine as substrates, which may point to a pivotal metabolic role in *M. tuberculosis* (Wink et al. 2013). Here, we describe the construction of a *M. tuberculosis* knockout (KO) strain for *iunH* gene, in vitro growth studies, and the effect of *iunH* deletion in *M. tuberculosis* in non-activated and activated macrophage model of infection, comparing with *M. tuberculosis* H37Rv wild-type (WT) and complemented (CP) strains.

## MATERIALS AND METHODS

**Fig. 1:**
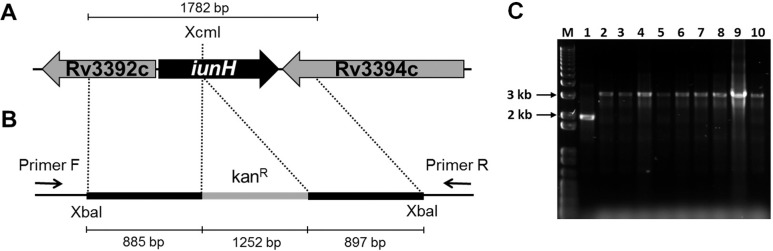
: genomic environment of *iunH* gene in *Mycobacterium tuberculosis* (A), regions cloned into pPR27*xylE* vector (B), and agarose gel electrophoresis of polymerase chain reaction (PCR) products from knockout strains (C). (A) Genomic region of *iunH* gene (927 bp) containing unique internal *XcmI* site and flanking genes; (B) the *iunH* gene and flanking regions (1782 bp) were amplified by PCR from *M. tuberculosis* H37Rv genomic DNA, and the *iunH* gene was disrupted by the insertion of a kanamycin cassette (kanR) into *XcmI* site (*iunH*::kanR). The *iunH*::kanR fragment was cloned into pPR27*xylE* vector using *XbaI* restriction site. Annealing regions of gene-specific screening primers forward (Primer F) and reverse (Primer R) for the possible knockout strains of *iunH* gene are indicated; (C) agarose gel electrophoresis of PCR products from knockout strains which were transformed with pPR27*xylE*::*iunH* kan. M - molecular marker 1 kb plus DNA Ladder (Invitrogen), PCRs were carried out with: 1 - *M. tuberculosis* H37Rv genomic DNA, Lanes 2 to 10 - possible knockout strains genomic DNA.

*Plasmid construction for generation of the knockout strain* - A fragment of 1782 bp containing the *iunH* gene (927 bp) with its flanking region (Fig. 1A) was amplified by polymerase chain reaction (PCR) from *M. tuberculosis* H37Rv genomic DNA, using primers forward (5’-tttttctagagcagcaggcgatgcgccagg-3’) and reverse (5’- tttttctagagacccgtcgccggcggtgc-3’), both containing *XbaI* restriction sites (underlined). The 1782 bp fragment was subsequently cloned into pUC19 using the *XbaI* restriction site. The *iunH* gene was disrupted by the insertion of a kanamycin cassette from pUC4K into unique internal enzyme restriction site *XcmI* (New England Biolabs, USA) (Fig. 1B). Insert was released from pUC19 derivative vector by digestion with *XbaI* (New England Biolabs, USA), and subcloned into *XbaI* linearised pPR27*xylE* vector (pPR27*xylE*::*iunH* kan) (Fig. 1B) (Pelicic et al. 1997).

*Plasmid construction for generation of the complemented strain* - The *iunH* gene flanked by about 200 bp upstream and 100 bp downstream, was amplified by PCR from *M. tuberculosis* H37Rv genomic DNA using primers forward (5’-ttttctagacagcgcgagatcgatcttg-3’) and reverse (5’-tttttctagacggtggtatctggagggaa-3’), both containing *XbaI* restriction sites (underlined), and was cloned into *XbaI* linearised pNIP40/b (pNIP40::*iunH*), a mycobacteriophage Ms6-derived integrative vector (Freitas-Vieira et al. 1998).

*Construction of the M. tuberculosis knockout strain* - Electrocompetent cells were prepared as described (Parish & Stocker 1998) with some modifications. *M. tuberculosis* H37Rv strain was grown in 50 mL of Middlebrook 7H9 (Becton Dickinson, BD, USA) 10% OADC (oleic acid, albumin, dextrose, and catalase) (BD, USA) 0.05% tween-80 (Sigma-Aldrich, USA) (liquid medium) to an OD_600_ of 0.6. Cells were washed two times in 0.05% tween-80, one time in 10% glycerol containing 0.05% tween-80, and were suspended in 500 µL of 10% glycerol containing 0.05% tween-80. Aliquots (200 µL) of fresh prepared competent cells were electroporated with approximately 2 µg of pPR27*xylE*::*iunH* kan plasmid in 0.2 cm cuvettes with a single pulse (2.5 kV; 25 mF; 1000 ohms). The pPR27*xylE* plasmid contains a thermosensitive origin of replication, the *xylE* reporter gene, and the *sacB* counterselectable marker. Bacteria were plated on Middlebrook 7H10 (BD, USA) 10% OADC (solid medium) containing 25 µg/mL kanamycin (Gibco, USA), and incubated at 32ºC. After six weeks, 1% pyrocatechol (Sigma-Aldrich, USA) was dropped on colonies to select those containing the plasmid. Three different yellow colonies were picked up from the transformant, and cultivated in liquid medium containing kanamycin 25 µg/mL at 32ºC. Individual cultures were plated on solid medium containing 25 µg/mL kanamycin, 2% sucrose (Fisher Scientific, USA), and cultivated at 39ºC. After four weeks, 1% pyrocatechol was dropped on colonies to select those that might be double crossover (DCO) strains. Nine white colonies were inoculated in liquid medium containing 25 µg/mL kanamycin, and cultivated at 37ºC for three weeks. Genomic DNA was isolated and PCRs were carried out using gene-specific screening primers forward (5’- ttcaggaaacgagcgaaggt-3’) and reverse (5’-gtgctatccggcggacac-3’) to determine whether the WT or the KO strain was present in the targeted chromosomal region (Fig. 1B).

*Construction of the M. tuberculosis complemented strain* - In order to obtain the CP strain with the *iunH* gene, the KO strain was transformed by electroporation with about 2 µg of the complementing plasmid construction, pNIP40::*iunH*. Electrocompetent cells were prepared as described above. Bacteria were plated on solid medium containing 50 µg/mL hygromycin (Invitrogen, USA) and 25 µg/mL kanamycin, and incubated at 37ºC. After three weeks, a single colony was cultivated in 5 mL of liquid medium with 50 µg/mL hygromycin 25 µg/mL kanamycin at 37ºC.

*Protein extraction* - WT, KO and CP strains were grown in 50 mL liquid medium containing the proper antibiotics until an optical density at 600 nm (OD_600_) of 0.5-0.7. Cellular pellets were washed twice using 10 mM Tris HCl pH 8.0. Cells were resuspended in 1 mL of the same buffer containing protease inhibitor cocktail (Promega, USA), and then transferred to 2 mL lysing matrix B tubes containing 0.1 mm diameter silica beads (MP Biomedicals, USA). Cells were disrupted using a L-Beader 3 (Loccus, Brazil) at a speed setting of 4000 rpm, 10 cycles of 30 s each, cooling between cycles. After lysis, the cell free supernatants were collected by centrifugation at 2300 x *g* for 10 min at room temperature. The supernatants were filtered through 0.22 mM Millex Durapore (Millipore, USA), and triton X-114 (Sigma-Aldrich, USA) extraction was carried out to obtain detergent and aqueous fractions as described previously (Malen et al. 2010).

*Western blot* - Anti-MtIAGU-NH mouse polyclonal antibody was produced by immunising a mouse with 50 mg of MtIAGU-NH purified protein (Wink et al. 2013) containing Freund’s incomplete adjuvant (Sigma-Aldrich, USA) (total volume of 100 µL) by subcutaneous route, followed by a booster injection after one month. After one more month, mouse was euthanised by deep isoflurane inhalation, and blood was collected by the descendant aorta. Serum was separated by centrifugation at 10,000 x *g*, for 10 min, aliquoted, and storage at -80ºC. Mycobacterial proteins from detergent fraction (50 mg) were loaded on sodium dodecyl sulphate 12% polyacrylamide gels (SDS-PAGE), and transferred to nitrocellulose membranes (iBlot Invitrogen, USA). Blots were blocked with 5% non-fat dried milk (Santa Cruz Biotechnology, USA) 0.05% tween-20 (Sigma-Aldrich, USA) in TBS (T-TBS), and probed with anti-MtIAGU-NH polyclonal mouse antibody in a 1:500 dilution. Membranes were washed three times with T-TBS, and alkaline phosphatase-conjugated anti-mouse secondary antibody (Invitrogen, USA) was used at a dilution of 1:5000. Chemiluninescent substrate (Novex by Life Technologies, USA) was used for detection with ChemiDoc (Bio-Rad, USA).

*LC-MS/MS* - The presence of MtIAGU-NH in the WT, KO and CP strains was also investigated by liquid chromatography coupled to mass spectrometry (LC-MS/MS) of SDS-PAGE slices. Sections from 28-40 kDa of each lane/strain were excised and submitted to in-gel digestion (Shevchenko et al. 2006). LC-MS/MS of peptides was performed on an Eksigent nanoLC Ultra 1D plus with AS-2 autosampler, coupled to a LTQ-XL Orbitrap Discovery (Thermo Scientific, USA). Peptide mixtures from SDS-PAGE slices were separated in reversed phase gradients in C18 (5 µM ODS-AQ C18 Yamamura Chemical Lab) column. Samples were analysed in technical triplicates. Mass spectra were acquired in a top 8 data-dependent manner with dynamic exclusion applied, and were searched against a non-redundant *M. tuberculosis* database for candidate peptides with the software Comet (Eng et al. 2012) in the platform PatternLab for Proteomics (Carvalho et al. 2016). The validity of the peptide spectra matches was assessed using the module Search Engine Processor from Patternlab for Proteomics, with a false discovery rate of 1%. Identified peptides from MtIAGU-NH were manually validated and peak areas of selected peptides were calculated with Skyline (MacLean et al. 2010).

*In vitro characterisation* - Growth curves were determined in Sauton’s medium containing 0.025% tyloxapol (Sigma-Aldrich, USA), in duplicates, at 37ºC, 80 rpm until reach the stationary phase. Aliquots were removed from each culture at different time points and the OD_600_ was determined. The data were evaluated with the two-way ANOVA analysis, followed by Bonferroni’s post-test, using GraphPad Prism 5.0. Differences were considered significant at the 95% level of confidence. In order to quantify the intracellular concentration of the bases (uracil, guanine, adenine, and hypoxanthine) and nucleosides (uridine, guanosine, adenosine, and inosine) in the WT, KO and CP strains, bacteria were grown in Sauton’s medium until an OD_600_ of 0.6 - 1.0. Cells were washed twice with 1x PBS, and autoclaved at 121ºC for 30 min, followed by sonication. An HPLC method (Dionex Ultimate 3000, Thermo Scientific, USA) to identify and quantify bases and nucleosides was modified from Wink et al. (2013). Solution A (1% acetic acid) was maintained at 100% for 20 min, followed by a linear gradient of 2 min up to 80% B (20 mM ammonium acetate) and 20% C (methanol and acetonitrile 1:1, v/v) for 10 min. The absorbance was measured at 262 nm, injection volume was 50 µL, 0.5 mL/min, and measurements were performed in triplicates.

*Macrophage infection* - Macrophage infection experiments are often used to determine mycobacterial strains virulence (Copenhaver et al. 2004, Katti et al. 2008). RAW 264.7 macrophage cell line was cultured in DMEM (Gibco, USA), supplemented with 10% heat inactivated fetal bovine serum (FBS) and 1% penicillin-streptomycin at 37ºC with 5% CO_2_. Before infection procedures, macrophages were seeded in 24-well culture plates at a density of 10^5^ cells per well in DMEM medium with 10% FBS and incubated for 24 h at 37ºC with 5% CO_2_. Two independent experiments were performed, one with non-activated and the other with activated macrophages, which were activated by the addition of 5 ng/mL of interferon-g (IFN-g) (R&D systems, USA) during 24 h. Infection of RAW 264.7 cells with WT, KO and CP strains was performed at a multiplicity of infection of 2:1 (bacteria/macrophage) at 37ºC with 5% CO_2_. After 18 h, infection was terminated by removing the overlaying medium, and each well was washed twice with sterile 0.9% NaCl solution to remove extracellular bacteria. At 18 h after infection, and two, three, six, seven and 10 days of incubation, wells were washed with sterile 0.9% NaCl solution, and the infected macrophages were then lysed with 0.025% SDS (Rodrigues Jr et al. 2014). DMEM media was changed after every three days of incubation. Lysates were serially diluted and plated on solid medium. Bacterial colony formation was evaluated after incubation of plates for three weeks at 37ºC. These experiments were performed in triplicates of each time point. The results were expressed as mean numbers of the logarithms of CFU per well, and were evaluated with the two-way ANOVA analysis, followed by Bonferroni’s post-test, using GraphPad Prism 5.0. Differences were considered significant at the 95% level of confidence.

## RESULTS AND DISCUSSION

*Construction of the M. tuberculosis knockout and complemented strains* - Among the nine clones screened for the KO of *iunH* gene, all suffered a double-crossover gene replacement event (Fig. 1C). To construct the CP strain, the pNIP40::*iunH* construction was transformed in the KO strain. The stability of the mutation introduced by gene replacement in *M. tuberculosis* was evaluated by plating KO and CP strains on media with and without antibiotics. The difference between the colonies obtained on plates containing antibiotics was not significant when compared with the ones obtained on plates without antibiotic, which indicates that the introduced mutation is stable (data not shown).

**Fig. 2:**
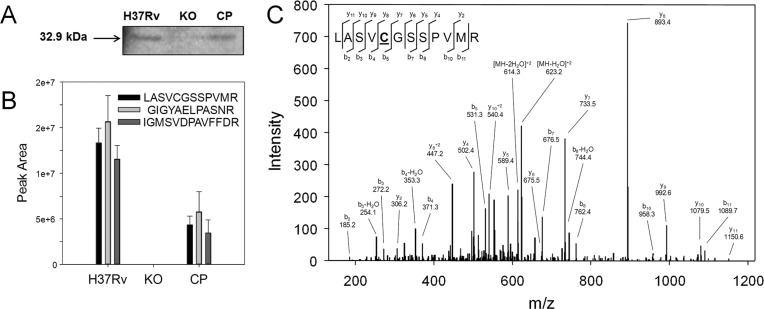
: evaluation of MtIAGU-NH expression. (A) Western blot analysis of protein extracts (50 mg) from detergent fractions of wild-type H37Rv (H37Rv), knockout (KO), and complemented (CP) strains. Bands were detected by incubation with anti-MtIAGU-NH polyclonal antibody (1:500) followed by alkaline phosphatase-conjugated anti-mouse secondary antibody (1:5000); (B) peak areas of peptides LASVCGSSPVMR (*m/z* 632.3128+2), GIGYAELPASNR (*m/z* 624.3226+2) and IGMSVDPAVFFDR (*m/z* 543.8024+2) were calculated using Skyline. LC-MS/MS analyses were performed 28-40 kDa section of SDS-PAGE samples. MS/MS spectra of identified peptides were manually validated; (C) representative MS/MS spectrum of the peptide LASVCGSSPVMR. The peptide was identified in both WT and CP samples. Doubly charged parent ion with neutral loses ([MH-H2O]+2 and [MH-2H2O]+2) and fragment b- and y-ions and their neutral loses are indicated. The cysteine (underlined) is carbamidomethylated.

*Evaluation of iunH knockout at protein level* - As shown in Fig. 2A, a 32.9 kDa band was detected by Western blot assay in extracts from WT and CP strains but was absent in the KO strain. The identity of the protein was further confirmed by LC-MS/MS from SDS-PAGE slices (28-40 kDa). Spectra matching MtIAGU-NH peptides were identified in WT and CP but not in KO extracts (Fig. 2B), confirming that the protein observed in WT and CP extracts by western blot is in fact MtIAGU-NH. As shown in Fig. 2B, the areas of the peptides identified on the WT sample are approximately 3-fold higher than the areas identified on CP sample. No peptides were identified on the KO sample; consequently peak areas could not be calculated. Fig. 2C shows a representative MS/MS spectrum of the peptide LASVCGSSPVMR, identified in both WT and CP samples. These results indicated that the disruption of *iunH* gene abolishes the expression of MtIAGU-NH protein in KO strain, and the MtIAGU-NH protein expression was restored in CP strain.

**Fig. 3:**
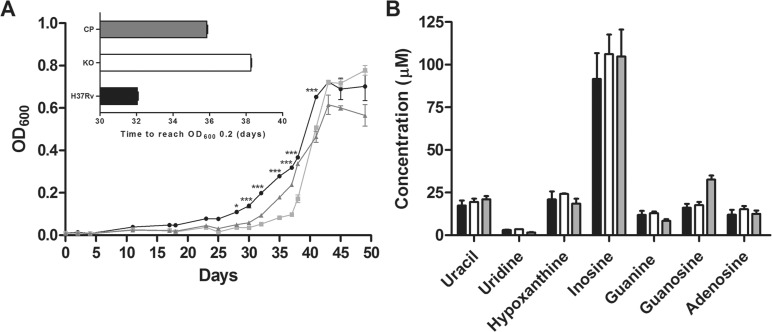
: in vitro characterisation of *iunH* knockout strain. Growth curve of WT (circle), KO for *iunH* gene (square), and CP (triangle) *Mycobacterium tuberculosis* strains grown in Sauton’s medium (A); to start growth curves the different strains were diluted to reach an OD600 of 0.01. Inset shows the time required for each strain to reach an OD600 of 0.2. Asterisks represent significant differences between WT and KO strains by the Bonferroni post-test, *p < 0.05, ***p < 0.001. Intracellular concentrations of bases (uracil, guanine, adenine, and hypoxanthine) and nucleosides (uridine, guanosine, adenosine, and inosine) in WT (black bar), KO (white bar), and CP (grey bar) *M. tuberculosis* strains grown in Sauton’s medium (B); adenine was not detected, and it was not included in graph; all measurements were performed in triplicates.

*In vitro characterisation* - The growth rate of the WT, KO, and CP strains were compared to determine whether *iunH* disruption lead to alterations during in vitro cultivation. Sauton’s is a defined medium that was used for in vitro characterisation experiments to avoid interferences with traces of bases and nucleosides from 7H9 medium. As shown in Fig. 3A, the deletion of *iunH* gene led to a delay of growth in Sauton’s medium during log phase. The *iunH* KO strain entered the early log phase of growth (OD_600_ of 0.2) six days later than WT strain (inset in Fig. 3A). No significant differences were found during lag and stationary phases of growth between WT and KO strains (Fig. 3A). Two independent experiments were performed, and similar results were obtained. The intracellular concentration of the bases and nucleosides was quantified in the WT, KO and CP strains. Uracil, guanine, adenine, hypoxanthine, uridine, inosine, guanosine and adenosine presented retention times of 10.53, 13.35, 14.34, 16.98, 19.89, 29.51, 29.64, and 30.36 minutes respectively. The calibration curves for all analyses were made from 0.48 to 62.5 µM and all presented a correlation coefficient above 0.99. No significant difference was observed in intracellular concentrations of either bases or nucleosides among the three strains when grown in Sauton’s medium (Fig. 3B). The absence of accumulation of purine bases and nucleosides in *iunH* KO strain could be explained by the redundancy found in nucleotide metabolism of *M. tuberculosis*. There are at least five enzymes in nucleotide salvage pathway that use MtIAGU-NH substrates or products: purine nucleoside phosphorylase (Rv3307), hypoxanthine-guanine phosphoribosyltransferase (Rv3624c), adenine phosphoribosyltransferase (Rv2584c), uracil phosphoribosyltransferase (Rv3309c), and pyrimidine nucleoside phosphorylase (Rv3314c) (Ducati et al. 2011, Villela et al. 2011); which might compensate for the absence of *iunH* gene in KO strain.

**Fig. 4:**
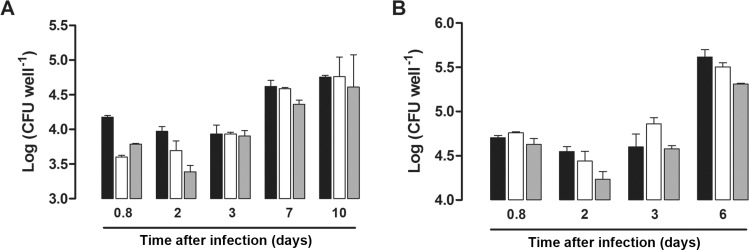
: infection of non-activated (A) and interferon-g activated (B) murine macrophages with the wild-type H37Rv (black bar), knockout (white bar) and complemented (grey bar) strains from *Mycobacterium tuberculosis*. The data are expressed as mean numbers of the logarithms of CFU per well of each strain of three independent measurements.

*Macrophage infection* - To examine whether the *iunH* gene was important for invasion and growth in phagocytic cells, we determined the bacterial loads of the WT, KO and CP strains by using the macrophage model of infection. Non-activated macrophages were infected with 1.4 x 10^5^, 2.2 x 10^5^, and 2.0 x 10^5^ CFU, while activated cells were infected with 2.6 x 10^5^, 2.5 x 10^5^, and 2.6 x 10^5^ CFU of WT, KO and CP strains, respectively, as determined at the day of infection. As shown in Fig. 4A, no significant difference was observed in intracellular growth among WT, KO, and CP strains in non-activated macrophages after 18 h, two, three, seven and 10 days after infection. Similar results were obtained with IFN-g activated macrophages, where no significant difference in bacterial load of KO strain was observed when compared with both WT and CP strains after 18 h, two, three and six days after infection (Fig. 4B). The disruption of *iunH* gene does not affect the *M. tuberculosis* growth in both non-activated and IFN-g activated RAW 264.7 cells. The concentration of 5 ng/mL of IFN-g or lower was shown to cause the activation of the endocytic pathway during the immune activation of RAW 264.7 (Pei et al. 2015), to induce the upregulation of the pro-inflammatory cytokine IL-18 in RAW 264.7 mouse macrophages (Kim et al. 2000), and to lead to its own production in mouse peritoneal macrophages (Di Marzio et al. 1994). In order to evaluate cytokine expression by non-activated macrophages infected with WT, KO and CP strains, the supernatants from 18 h, and three days post infection were collected and interleukin (IL)-1b, tumor necrosis factor (TNF)-a, and IFN-g were quantified by ELISA using a commercial kit (data not shown). TNF-a and IL-1b levels of expression were not significantly different among groups, while IFN-g expression was not detected in culture medium of cells infected with all strains (data not shown).

In this work, we constructed a *M. tuberculosis* KO strain for *iunH* gene, validated the gene deletion at protein level, characterised the KO strain in vitro, and evaluated its ability to invade and grow in non-activated and IFN-g activated macrophages. The absence of accumulation of purine bases and nucleosides in *M. tuberculosis iunH* KO strain in Sauton’s medium, together with the fact that *iunH* gene is not important for *M. tuberculosis* virulence in macrophages, could be explained by the redundancy found in nucleoside/nucleotide metabolism of *M. tuberculosis*. As mentioned previously, there are at least five enzymes in nucleotide salvage pathway from *M. tuberculosis* that use MtIAGU-NH substrates or products (Ducati et al. 2011, Villela et al. 2011). These enzymes might compensate for the absence of *iunH* gene in KO strain, consequently maintaining the nucleotide pool within the cell. Our results indicated that MtIAGU-NH is not a target for drug development.
